# Carbon utilization and storage through rehabilitation of groundwater wells

**DOI:** 10.1038/s41598-024-64135-z

**Published:** 2024-06-15

**Authors:** Vivek V. Patil, Gabriella Basso, Steven Catania, Christopher Catania, Timothy Ostapuk, Robert Vince

**Affiliations:** 1 WSP USA Inc., South Jordan, UT 84095 USA; 2 Subsurface Technologies Inc., Rock Tavern, NY, 12575 USA; 3 WSP USA Inc., Phoenix, AZ 85034 USA; 4 WSP USA Inc., Helena, MT 59601 USA

**Keywords:** CO$_{2}$ sequestration, CCUS, Groundwater, Well rehabilitation, Climate change, Preventive well maintenance, Climate-change mitigation, Hydrology, Environmental impact, Hydrogeology, Projection and prediction

## Abstract

According to the Intergovernmental Panel on Climate Change (IPCC) of the United Nations (UN), rise in atmospheric concentration of carbon dioxide (CO${_2}$) due to anthropogenic factors is considered as the primary driver for global climate change. With almost every major corporation around the world working towards their “net-zero goals”, it is becoming increasingly important to have more technologies that can help reduce carbon footprint. Achieving sequestration of CO${_2}$ in the subsurface through Carbon Capture Utilization and Storage (CCUS) technologies like CO${_2}$-Enhanced Oil Recovery, CO${_2}$-Enhanced Geothermal Systems, CO${_2}$-Enhanced Coal Bed Methane, etc. is well accepted. We introduce yet another attractive CCUS opportunity through well rehabilitation. Aqua Freed^®^ and Aqua Gard^®^ are well-known well rehabilitation and preventive well maintenance technologies that utilize (inject underground) liquid CO${_2}$ for the purpose. The goal of this study was to quantify the storage capacity of Aqua Freed^®^ and Aqua Gard^®^, and establish their CCUS credentials. Depending on the well being serviced, these technologies can inject up to 40 US tons of CO${_2}$ per well. Based on field data collection and statistical modeling, we estimated that 82–96% (median 90%) of the injected CO${_2}$ remains in the subsurface post injection. Overall, our results and analysis of the US market suggest that using CO${_2}$ for well rehabilitation and maintenance has a storage potential of several megatonnes of CO${_2}$ annually in the US alone.

## Introduction

Rise in carbon dioxide (CO${_2}$) concentration in the atmosphere due to anthropogenic activities is considered as one of the primary drivers for climate change^1,2^. The need to neutralize the carbon footprint and move towards ‘net-zero’ emissions is being widely recognized^3,4^. The Intergovernmental Panel on Climate Change (IPCC) has reported that limiting global warming to 1.5 °C above the pre-industrial temperatures will require reaching net-zero emissions globally by 2050^5^. Most of the major corporations in the world have accordingly set their ‘net-zero’ goals, which means their own timeline to becoming carbon neutral^6,7^. These ambitious goals can be achieved by (1) transitioning to low-carbon technologies and ‘green’ energy sources^8,9^, and/or (2) carbon offsetting, which means removing a quantity of CO${_2}$ equivalent to one’s carbon footprint^10^.

One of the primary ways of carbon offsetting is by capturing CO${_2}$, either from effluent industrial streams or directly from air, and keeping it away from the atmosphere by its reuse, recycle, storage and eventually sequestration^11^. While there are several ways to achieve sequestration, both naturally and technologically (e.g., oceanic, rock weathering, mineralization, geologic, etc.), the most promising of these methods in terms of storage capacities, timescale and environmental impact is considered the geologic sequestration of CO${_2}$^12^.

Before storing it away, utilizing and recycling the CO${_2}$ is often desirable and more feasible from a commercial standpoint^13^. Carbon Capture Utilization and Storage (CCUS) is a bracket term used to refer to any technological process that captures, utilizes, and stores the CO${_2}$ away from the atmosphere^14^. The most widely known CCUS technology is CO${_2}$-Enhanced Oil Recovery (CO${_2}$-EOR), wherein CO${_2}$ is injected into the subsurface to aid the tertiary recovery of crude oil that may be otherwise difficult to produce^15^. Other known CCUS technologies include CO${_2}$-Enhanced Geothermal Systems (using CO${_2}$ as a working fluid instead of water^16^) and CO${_2}$-Enhanced Gas Recovery (using CO${_2}$ to enhance natural gas recovery from the subsurface^17^). All these technologies involve injecting CO${_2}$ into the subsurface for a technological and commercial advantage. In the process, a portion of the CO${_2}$ inadvertently remains in the subsurface.

In this paper, we introduce the use of CO${_2}$ for rehabilitation and preventive maintenance of groundwater wells as another CCUS opportunity. All groundwater wells develop mineral and biological build-up over time^18^, which results in a decline in the well’s capacity to produce water (specific capacity) and the water quality. The traditional approach taken by well owners has been to rehabilitate a well when its specific capacity has dropped significantly. However, studies have shown that preventive maintenance scheduled on an annual basis helps increase the longevity of the well components, maintain better water quality, and reduce long-term operational costs^19^.

The benefits of using CO$_2$ on improving performance and aiding maintenance of wells have been recognized by the Oil & Gas sector. For example, Wilson and Bell^20^ have proposed a method of a combination of liquid CO$_2$, alcohol and surfactants for cleaning up wellbore and near-wellbore areas of gas wells. In addition to well cleanup, CO$_2$ has also shown to provide benefits when used as a fracturing fluid for stimulation of low-permeability gas reservoirs^21–23^.

The technology of using CO$_2$ for groundwater well rehabilitation and maintenance has been established through the Aqua Freed^®^ and Aqua Gard^®^ processes^24,25^ that have been applied to groundwater well systems across the US and in some parts of Europe for over 35 years. Utilizing CO${_2}$ for well rehabilitation has proven to be advantageous over other methods (e.g., traditional chemical and acid treatment, wire charge methods, fluid percussive methods, etc.) in terms of the well performance as well as the impact on water quality^26^. The goal of this study was to quantify the potential of carbon utilization and storage via CO${_2}$-powered well rehabilitation technologies, using the example of Aqua Freed^®^ (AF) and Aqua Gard^®^ (AG), with field-collected data analysis and modeling. Using field measurements and statistical modeling approaches, we first estimate the percentage of injected CO$_2$ to get stored in the aquifer for a single well based on a data from a selected set of wells. We then apply these estimates to a larger database to estimate the total CO$_2$ that has been stored over the past decade through these technologies. Finally, we estimate the storage potential of this CCUS technology based on the US market size of private and public groundwater wells.

### CO${_2}$-powered well rehabilitation process

Rehabilitation and preventive maintenance of groundwater supply wells using CO$_2$ has been performed by Subsurface Technologies Inc. (STI) by using Aqua Freed^®^ (AF) and Aqua Gard^®^ (AG) respectively^24,25^. The method involves controlled injection of CO${_2}$ (gaseous and liquid phases) at specific pressures and temperatures, which lends significant energy near the downhole area, resulting in the detachment, dissolution and removal of sediments and encrustation within the well screen and the surrounding aquifer. The general steps in the process are as follows:

1. A pre-injection pump test is conducted to determine the performance of well prior to treatment. It provides data on how the well and pump performs pre-treatment. A video inspection of the well is conducted to determine proper packer placement.
2. A combination of gaseous and liquid CO$_2$ is injected through the packer into the aquifer at various temperatures and pressures, resulting in expansion and energy release.
3. After the injection is complete, the system is allowed to rest for a certain amount of time (usually around 12 hours) for the treatment to work.
4. A small of amount CO$_2$ gas accumulated around the well-head is vented out (released to the atmosphere).
5. Since the groundwater around the well has high CO$_2$ concentration and an acidic pH, the well is flushed (groundwater is produced) until the CO$_2$ concentration and pH return to background values. The CO$_2$-laden groundwater is discarded as wastewater. The time and flow rate of flushing vary for each well and are determined by the field experts depending on the field and subsurface conditions.
6. Lastly, a post-injection pump test is conducted to determine the well performance post treatment.

**Figure 1:**
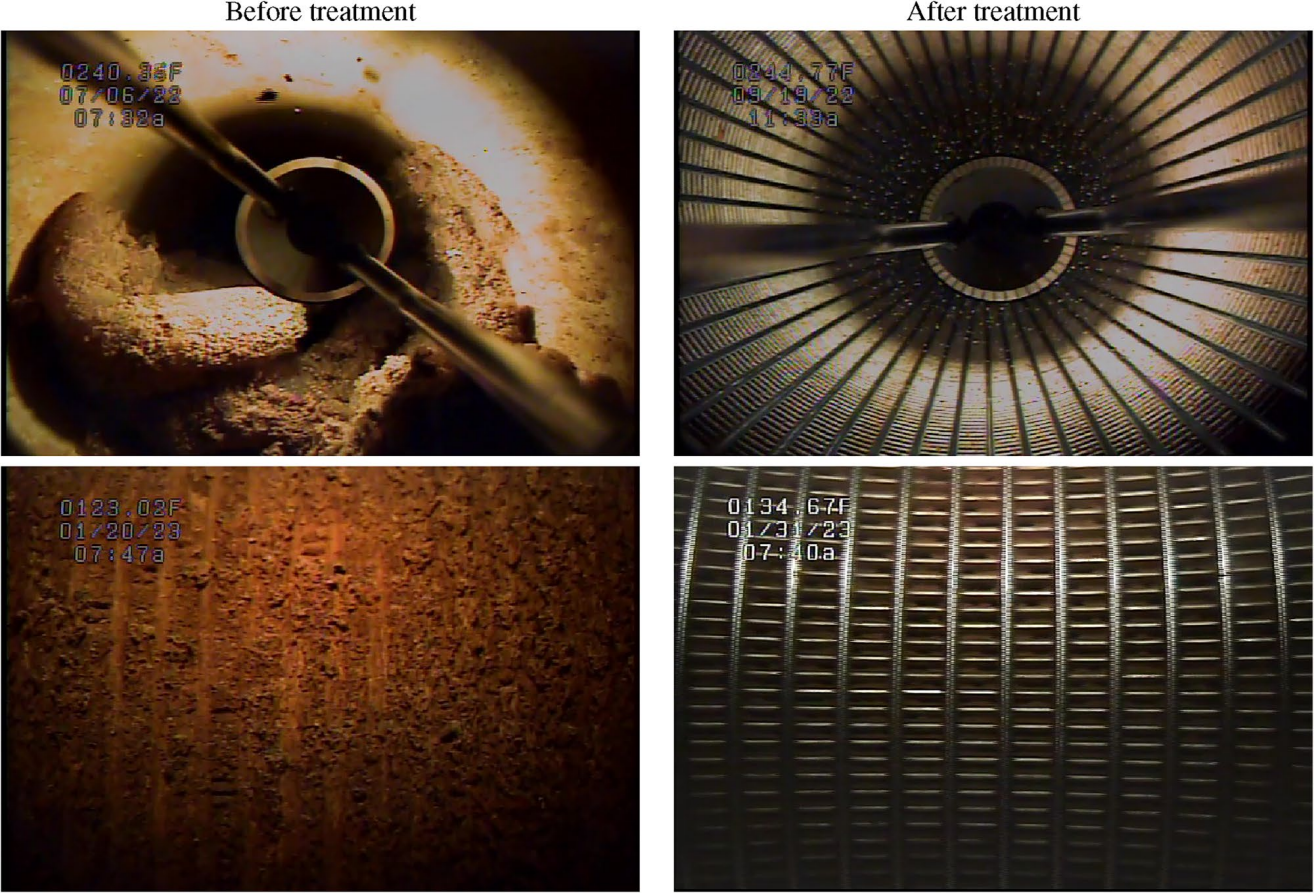
Images of well screens captured during in-situ video inspections conducted before and after treating a well with CO$_2$.

Figure 1 presents in-situ photographs of the inside of a groundwater well before and after its AF treatment. The photographs clearly demonstrate the extent of mineral, biological and iron incrustation build-up. CO$_2$-powered AF rehabilitation treatments have shown to increase a well’s specific capacity by as much as 20 times^26^.

The quantity of CO$_2$ injected depends on the size of the well and how deep the well is. While the exact quantity for a particular well is based on a proprietary equation that takes into account well depth, well diameter, and borehole/screen length, the typical values are 2.5–5, 6–12 and 20–40 US Tons of CO$_2$ for small, medium, and large-sized wells respectively.

Both AF and AG follow the same above process. The main difference between the two is that AG, being a preventive care program for a well that is a follow up after AF treatment, requires a smaller quantity of CO$_2$ as compared to AF (typically about one-fifth) for the same well.

### Approach to estimating storage potential

Since the quantity of injected CO$_2$ is known for a well, the foremost way to quantify how much of it got stored is by estimating the loss of CO$_2$ at each of the steps of the AF/AG process described above. Subtracting the total loss of CO$_2$ throughout the process from the injected quantity directly leads us to how much CO$_2$ remained in the subsurface after the process was completed and the well was handed back to the owner.

An up-close examination of the process led to the conclusion that any losses of CO$_2$ would happen at the venting, flushing and post-injection pump test. Usually, the post-injection pump test is directly followed by flushing. Therefore, the loss during the post-injection pump can be combined with the loss during flushing. Thus, the mass balance equation of CO$_2$ for the AF/AG process is

1 $$\begin{aligned} \text {CO}_2\,\text {stored} = \text {CO}_2\,\text {injected} - \{\text {CO}_2\,\text {loss during venting}+\text {CO}_2\,\text {loss during flushing}\} \end{aligned}$$

## Results

### Loss estimation

#### Venting

Data during the venting process was collected using a flow meter at 2 independent wells undergoing AG treatment. The venting time for the first well was 1 minute at a flowrate of 100 ft$^3/$min for first 30 seconds and 25 ft$^3/$min for the remaining 30 s. Converting the volume of CO$_2$ to mass assuming CO$_2$ density of 1.87 kg/m$^3$ (at $\sim$15 $^{\circ }$C and 1 atm), the mass of CO$_2$ lost during venting was estimated to be 7.2 lbs. Given the mass of CO$_2$ at this well was 2000 lbs, the percentage of CO$_2$ lost during venting was estimated to be 0.36%. Following the same procedure, the percentage of CO$_2$ lost during venting at the second well was estimated to be 0.39%. The timing of the venting at other wells was observed to be in the similar range. Therefore, it was assumed that the loss of venting is expected to be less than 1% in all cases and can be ignored in the storage estimations.

#### Flushing (including post-treatment pump test)

**Figure 2:**
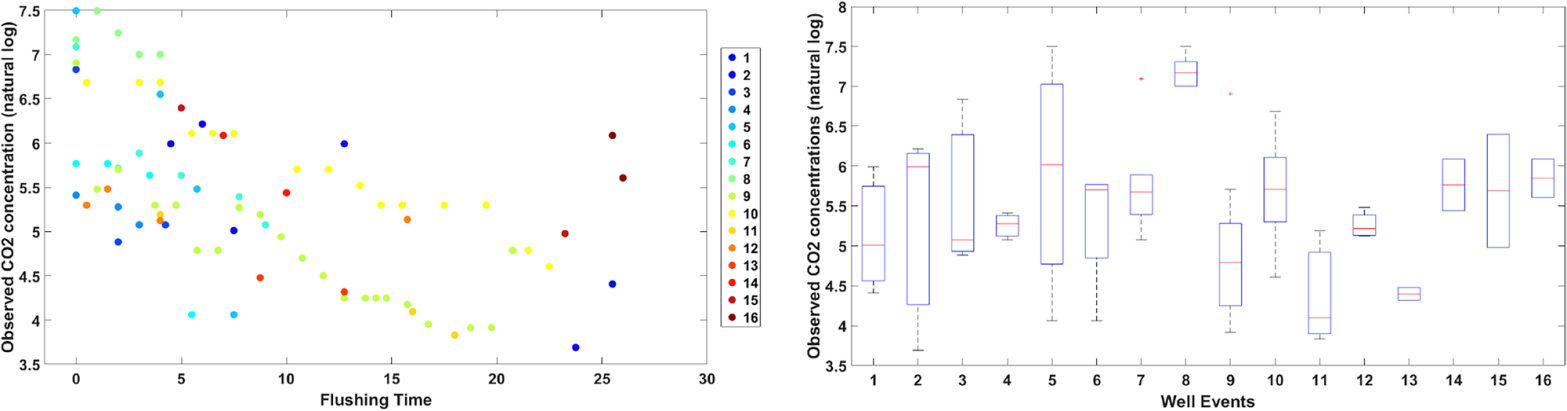
Observed CO$_2$ concentrations during flushing of 16 well events (dataset1).

As part of this study, CO$_2$ concentrations and pumping rates were noted during flushing at 16 wells being processed with AF/AG (referred to as dataset1). In addition, historic data for 15 additional wells was retrieved from STI’s records with pumping rate data but no available measurements of concentration (referred to as dataset2). Overall, these 31 wells represent a wide range of hydrological conditions (e.g., consolidated vs. unconsolidated aquifers, injection depths ranging from 40 to 560 feet below earth’s surface) and engineering details (e.g., open vs. screened hole, injection pressures ranging from 40 to 450 psi). Dataset1 (with 2 or more conentration data points for each well) was used to model the trend of concentration drop along time. A visual inspection of the concentration vs. time plots indicated an exponential trend in most of the wells. Therefore, a natural log of these values would follow a linear trend. The distribution of concentration data collected during flushing of 16 well events (dataset1) is presented in Fig. 2

**Figure 3:**
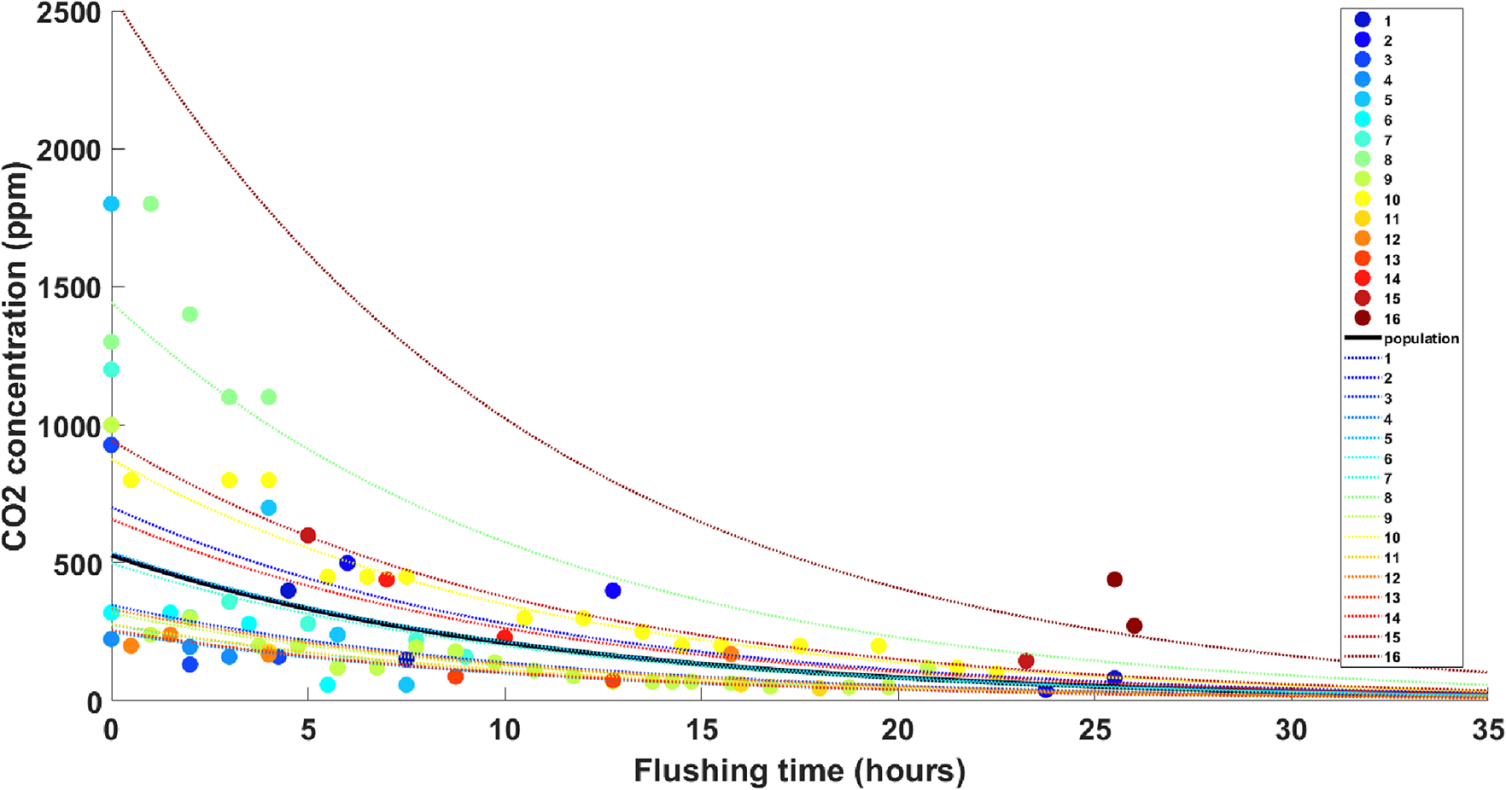
CO$_2$ concentration profile in mg/l or ppm along time during flushing for the 16 well events (dataset1). The color coded circles represent actual data points for each well and color coded lines represent the regression fits to these data points using the LME model (converted from natural log to real values). The thicker black line represents the ‘fixed’ effect or the population trend, while the colored dash lines represent the ‘random’ effects or the individual well trends.

**Figure 4:**
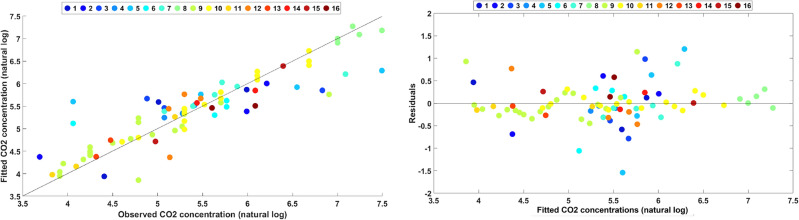
Plots showing the goodness of fit of the LME model to dataset1.

In order to determine a general trend and rate of concentration drop during the flushing process, a linear mixed-effects (LME) regression^27^ approach was chosen. This approach is widely used for multilevel or hierarchical modeling, wherein both the population-wide trend as well as trends at the level of subgroups are important^28,29^. In our case, the LME model takes into account both invidual well trends (called ‘random’ effects) as well as a population-wide trend (called ‘fixed’ effect). Detailed discussion on model selection and cross-validation is featured in the “Methods” section and supplementary information. The relevant statistics of the LME regression model fitted to dataset1 are presented in Table 1. Figure 3 presents the resulting regression fits for the individual wells (random) and the population fit (fixed) converted back from natural log values to real values of concentration. The goodness of this fit is graphically presented in Fig. 4. The fitted vs. observed values plot shows that the points align uniformly along the diagonal (y = x), which signifies that model predictions compare closely with observed data. The residual plot shows that the points are scattered randomly around the residual = 0 line with no particular pattern, which indicates the LME model is the correct choice for this dataset.

**Table 1:** The results of the Linear Mixed-Effects (LME) model fitting to the CO$_2$ concentration trends during flushing for 16 individual wells (dataset1).

Fixed effects (population)				Random effects (individual wells)	
Parameter	Estimate (std. error)	P-value	95% CI	Parameter	Std. dev. (lower, upper)
Intercept	6.271 (0.201)	1.75E−46	5.87 to 6.672	Intercept PDF	0.694 (0.457, 1.05)
Slope	− 0.092 (0.009)	5.21E−16	− 0.11 to − 0.074	Slope PDF	Does not vary

Using the individual concentration trends for dataset1, the population concentration trend for dataset2, and known volumetric flowrates during flushing for these wells, the mass of CO$_2$ lost incrementally along time is simply calculated as a mathematical product of concentration (mass per volume), flowrate (volume per time) and flushing time. This yields us the total mass of CO$_2$ lost during flushing, which when compared to the mass of CO$_2$ injected leads to the effective percentage of CO$_2$ stored in the subsurface for that well. The estimated percent of CO$_2$ stored for each of the total 31 wells from dataset1 and dataset2 are presented in Table 2.

**Table 2:** A database of 31 AF/AG events used for calculating the loss of CO$_2$ during flushing along with the estimated percentage of CO$_2$ stored during each event is presented in this table.

Well event	Event type	Aquifer type	Well type	CO$_2$ injected (US Tons)	%CO$_2$ stored
**1**	**AG**	**Consolidated**	**Open hole**	**1**	**80.8**
**2**	**AF**	**Consolidated**	**Open hole**	**3**	**97.6**
**3**	**AG**	**Unconsolidated**	**Screened**	**0.75**	**82.1**
**4**	**AG**	**Unconsolidated**	**Screened**	**0.75**	**87.3**
**5**	**AG**	**Unconsolidated**	**Screened**	**0.75**	**50.3**
**6**	**AG**	**Unconsolidated**	**Screened**	**0.75**	**85.3**
**7**	**AG**	**Unconsolidated**	**Screened**	**0.75**	**57.5**
**8**	**AG**	**Consolidated**	**Open hole**	**2**	**93.2**
**9**	**AF**	**Unconsolidated**	**Screened**	**1.5**	**97.5**
**10**	**AF**	**Unconsolidated**	**Screened**	**5**	**86.0**
**11**	**AF**	**Consolidated**	**Open hole**	**6**	**99.1**
**12**	**AF**	**Consolidated**	**Open hole**	**5**	**98.6**
**13**	**AF**	**Consolidated**	**Open hole**	**0.5**	**90.9**
**14**	**AG**	**Consolidated**	**Open hole**	**2**	**89.0**
**15**	**AG**	**Consolidated**	**Open hole**	**1**	**93.4**
**16**	**AF**	**Unconsolidated**	**Screened**	**1**	**10.3**
*17*	*AG*	*Consolidated*	*Screened*	*2*	*90.2*
*18*	*AG*	*Consolidated*	*Open hole*	*1.25*	*82.7*
*19*	*AG*	*Consolidated*	*Open hole*	*1.25*	*87.6*
*20*	*AG*	*Unconsolidated*	*Screened*	*0.375*	*46.2*
*21*	*AG*	*Unconsolidated*	*Screened*	*0.25*	*86.2*
*22*	*AG*	*Unconsolidated*	*Screened*	*2*	*95.9*
*23*	*AG*	*Unconsolidated*	*Screened*	*0.75*	*9.3*
*24*	*AG*	*Unconsolidated*	*Screened*	*0.75*	*42.8*
*25*	*AF*	*Unconsolidated*	*Screened*	*2.5*	*94.0*
*26*	*AF*	*Unconsolidated*	*Screened*	*4*	*95.2*
*27*	*AF*	*Unconsolidated*	*Screened*	*2*	*90.5*
*28*	*AF*	*Unconsolidated*	*Screened*	*3*	*93.6*
*29*	*AF*	*Consolidated*	*Open hole*	*5*	*97.5*
*30*	*AF*	*Consolidated*	*Open hole*	*2.5*	*95.8*
*31*	*AF*	*Consolidated*	*Open hole*	*8*	*97.8*

The first 16 well events (rows marked with ‘bold’), considered as dataset1, were conducted as part of this study and CO$_2$ concentration measurements along time were available along with flowrate information. The next 15 well events (rows marked with ‘italic’ ) were retrieved from STI’s records prior to this study and only flowrate information was available for these events.

### Storage estimation

#### Storage estimates per well

**Figure 5:**
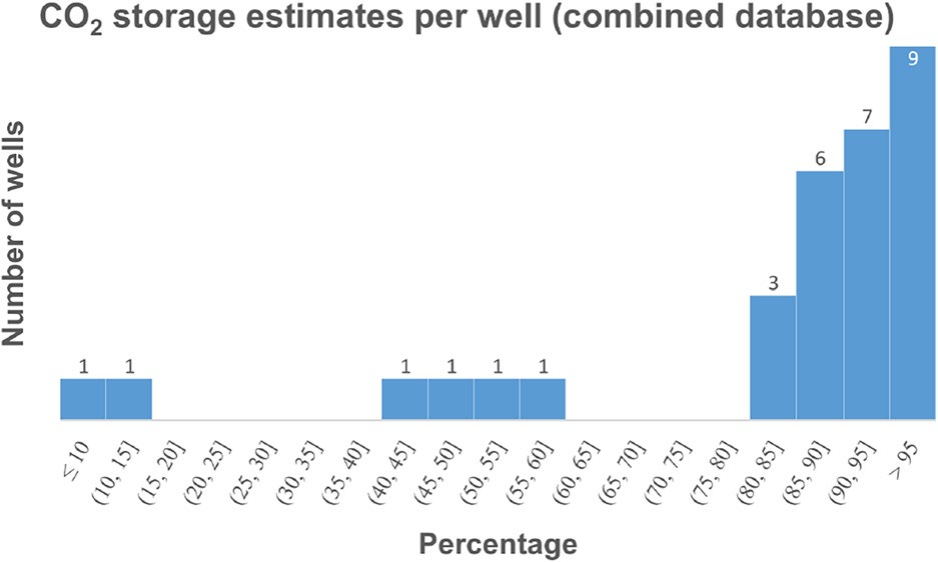
Histogram of the estimated percentage CO$_2$ stored by individual wells for the total 31 well events (dataset1 and dataset2 combined).

The general statistical distribution across the entire database of well events is presented in Fig. 5 and Table 3. Based on the combined set of 31 wells, the median estimated proportion of CO$_2$ that remains or is stored in the subsurface after any AF or AG process is about 90% and the interquartile range is between 82% to 96%. This means that at least three-fourth of all the groundwater well treatments with CO$_2$ via the AF/AG processes will store more than $\sim$82 percent (typically $\sim$90%) of the injected mass of CO$_2$ underground after the treatment process is complete.

**Table 3:** Statistics of the estimated percentage of CO$_2$ stored from the dataset of individual wells.

Estimated % CO$_2$ stored			
Dataset	1	2	Combined
# Wells events	16	15	31
Q0 (min)	10.3	9.3	9.3
Q1 (25th)	81.8	84.5	82.4
Q2 (median)	88.1	90.5	90.2
Q3 (75th)	94.4	95.5	95.5
Q4 (max)	99.1	97.8	99.1

Across the entire set of 31 wells, the median storage estimation is $\sim$90% with the interquartile range from $\sim$82 to 96%.

#### Storage estimates for the year 2021

**Figure 6:**
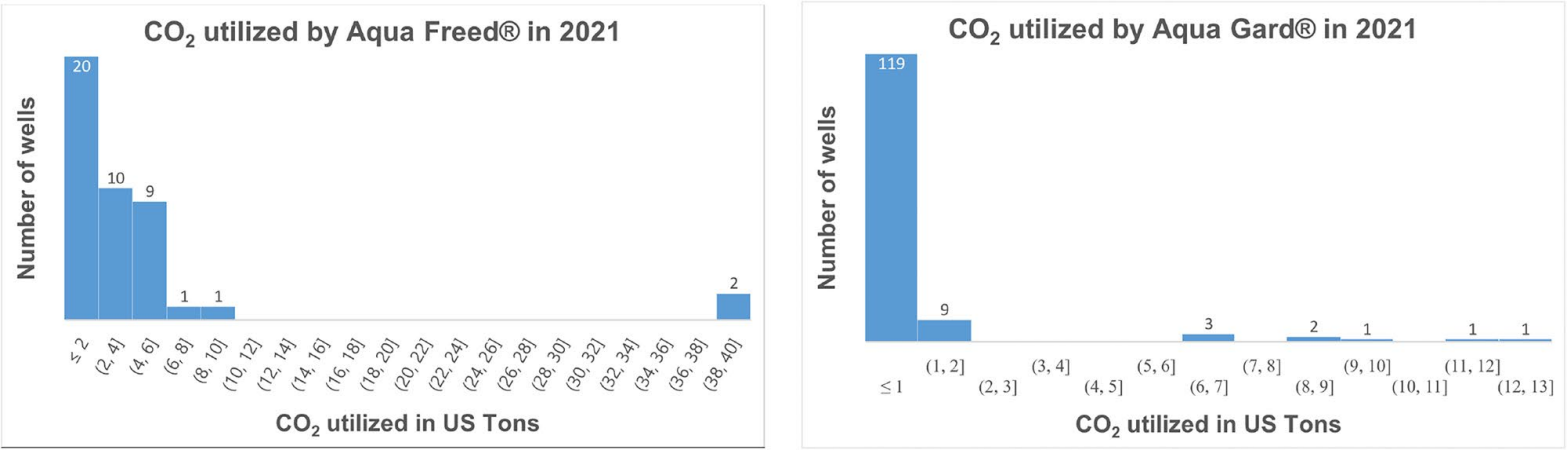
Statistical distribution (histogram) of the CO$_2$ utilized by STI’s operations in 2021 based on individual wells. Left histogram represents AF treatments and right histogram represents AG treatments.

The total quantity of CO$_2$ utilized (injected into the subsurface) by the well rehabilitation and maintenance services completed by STI in the year 2021 was 700,600 pounds or 350.3 US Tons. The statistical distribution based on individual wells serviced in 2021 for AF and AG treatments is shown in Fig. 6. Based on the interquartile range of the percent storage for individual wells calculated above, the estimated mass of CO$_2$ stored by STI’s operations in 2021 is 287 to 336 US tons. These estimates represent STI’s operations only and are not inclusive of any CO$_2$-powered well treatments conducted by the authorized providers of STI.

#### Storage estimates for the decade 2012-21

**Figure 7:**
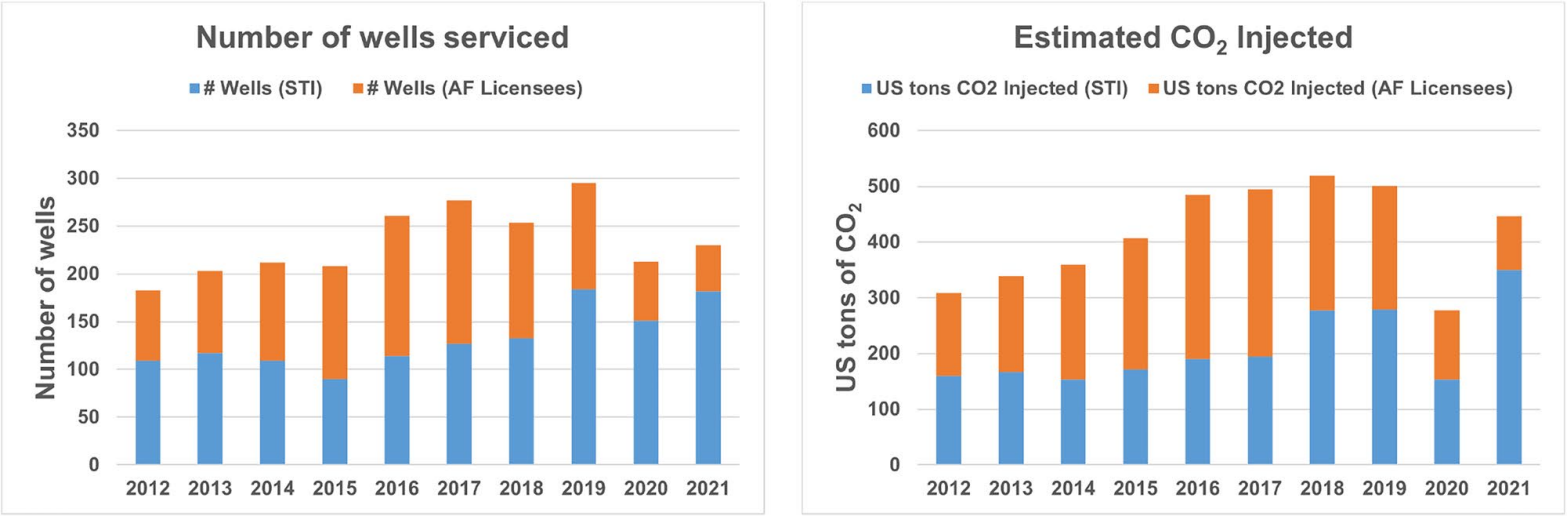
Graphical depiction of the yearly distribution of number of wells serviced and estimated CO$_2$ injected by STI and its authorized providers.

The total estimated CO$_2$ utilized by CO$_2$-powered well rehabilitation and maintenance treatments (AF and AG) in the past decade (2012-2021) was estimated by accounting for all the wells serviced directly by STI as well their authorized providers (licensees). To the best of our knowledge, there are no other entities operating such technologies for the purpose of rehabilitating groundwater wells. A yearly breakdown of the number of wells serviced and the tonnage of CO$_2$ utilized is presented in Table 4 and graphically represented in Fig. 7.

**Table 4:** A yearly distribution of CO$_2$-powered well rehabilitation and maintenance activities for the past decade (2012–2021).

Year	# Wells (STI)	# Wells (AF licensees)	# Wells (total)	US tons CO_2_ injected (STI)	US tons CO_2_ injected (AF licensees)
2012	109	74	183	160.1	148
2013	117	86	203	167.3	172
2014	109	103	212	153.6	206
2015	90	118	208	171.2	236
2016	114	147	261	191.0	294
2017	127	150	277	194.6	300
2018	133	121	254	277.3	242
2019	184	111	295	278.8	222
2020	151	62	213	153.6	124
2021	182	48	230	350.3	96
Total	1316	1020	2336	2097.6	2040

An estimated 4100 tons of CO$_2$ was utilized (injected into the subsurface).

**Figure 8:**
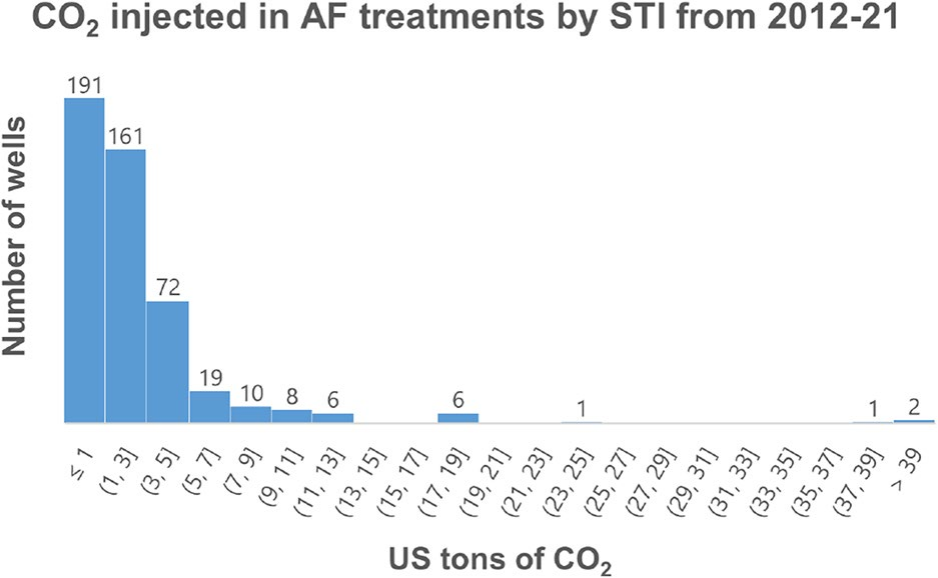
Histogram of the quantities of CO$_2$ injected in individual AF treatments done by STI from 2012-2021.

While the exact quantity of CO$_2$ injected at each well by STI in the past decade was known, there was no information on that for the AF authorized providers except the number of wells serviced. However, we can assume that the median and interquartile range of the CO$_2$ injection quantities for AF licensees would be similar to that of the AF treatments done by STI directly. Figure 8 shows the statistical distribution of the CO$_2$ quantity utilized in 477 AF treatments done by STI in the past decade. The median value of the distribution was 2 tons, with the interquartile range from 0.65 to 3.5 tons of CO$_2$. The estimated tonnage of CO$_2$ utilized by AF authorized providers in Table 4 was calculated based on the median value of 2 tons. The total range of CO$_2$ tonnage (based on the interquartile range of 0.65 to 3.5 tons) was estimated to be 2761 to 5668 tons of CO$_2$. Thus, assuming a median 90% of storage, the cumulative estimated CO$_2$ stored by STI and its authorized providers together over the past decade (2012-2021) is 2485 to 5101 US tons (median value of 3724 US tons).

#### Future projections

According to the National Ground Water Association (NGWA), there are a total of about 16 million water wells in the U.S.^30^ About 13 million of these wells are residential, while the rest are non-residential (industrial, public water supply, agricultural, etc.). Based on STI’s experience, residential wells are typically small wells that generally require about 1000 lbs of CO$_2$ for AF treatment. Non-residential wells can be divided into three categories, small, medium and large, based on their size and capacity. The quantity of CO$_2$ required for AF treatments is 2.5–5 tons, 6–12 tons and 20–40 tons for small, medium and large water wells respectively. Subsequent AG treatments for annual maintenance typically require about one fifth of that.

Based on the CO$_2$ requirements and STI’s database of processed wells from the past decade (see Fig. 8), the ratio of small, medium and large wells is 89%, 8% and 3%. This ratio is likely to be a conservative ratio, as this includes both residential (which are usually smaller in size and larger in number) and non-residential wells. However, due to lack of information, we assume the same ratio applies to non-residential wells across the U.S.

Based on the number of wells in the U.S. and the typical amount of CO$_2$ required for rehabilitation and annual maintenance, we estimate (as shown in Table 5) that adopting CO$_2$-powered well rehabilitation and maintenance techniques have the potential of utilization and storage of about 20 million US tons of CO$_2$ every 15 years (average rehabilitation cycle for a water well). Annual maintenance programs like AG can help store an additional 4 million US tons on an annual basis. These are the estimates of the utilization potential based on a single country.

**Table 5:** Estimating the national capacity of CCUS through rehabilitation and maintenance of water wells.

	Residential	Non-residential			Total
		Small	Medium	Large	
No. of water wells in the US	13,000,000	2,666,667	232,704	100,629	16,000,000
CO_2_ injected per well via AF (lb)	1000	7500	18,000	60,000	
Total CO_2_ injected via AF (million US tons)	6.5	10	2.1	3	21.6
Estimated CO_2_ stored via AF (million US Tons)	5.9	9	1.9	2.7	19.5
Annual CO_2_ stored via AG (million US Tons)	1.2	1.8	0.4	0.5	3.9

## Discussion

- *Storage vs. sequestration* It is important to note that the terms storage and sequestration are often used interchangeably, but there is a subtle difference between them. Storage is a broad term, of which sequestration can be considered a subset that usually refers to long-term, ‘permanent’ storage (typically hundreds to a thousand years in the future)^31,32^. The focus of this study was on the quantifying CO$_2$ storage (what remained in the subsurface after the process was completed and the well was handed back to the owner). Investigating long-term fate and migration of the injected CO$_2$ is a more complex scope of study and will require multiphase geochemically reactive transport modeling of site-specific case studies (similar examples of which can be found in the literature)^33–35^. Studies following this will aim at tackling these research directions.
- *Long-term fate of injected CO*$_2$ This study is the first study to explore the idea of injecting CO$_2$ into shallow groundwater aquifers for well-rehabilitation. The down-side of being a first of it’s kind project is lack of prior data for comparison. To be best of our knowledge, we have not come across another study where a direct comparison would be possible. Typically, once CO$_2$ is injected, it remains in the subsurface as either a separate gas/supercritical phase, dissolved aqueous phase or precipitated mineral phase. The common modes of CO$_2$ trapping in the subsurface are stratigraphic or structural gas trapping (being trapped as a gas phase between strategic geologic features like faults, seals, caprocks etc.)^36,37^, residual gas trapping (residual irremovable gas being trapped in fine pores by capillary forces)^38,39^, dissolution trapping (being trapped in solution as bicarbonate)^40,41^, and mineral trapping (being converted to a carbonate mineral phase)^42,43^. The relative proportion of CO$_2$ stored via each of these individual mechanisms will depend on the exact, site-specific hydrogeological conditions^44^. For the present application, because of relatively low quantities of CO$_2$ being injected compared to CO$_2$-EOR or deep saline sequestration, it can be expected that the structural gas trapping capacity will be relatively low. Almost all of the stored CO$_2$ will get converted to aqueous CO$_2$ relatively instantaneously. Geochemical buffering of the elevated acidity from this solubility trapping by in-situ water rock reactions will consume the dissolved CO$_2$ and may convert some of it to secondary precipitated minerals. Initially, when the fluid pressures are high, especially near the well, CO$_2$ will dissolve in water forming bicarbonate. As pressures reduce, the bicarbonate may convert back to gaseous CO$_2$, especially at near-surface depths^45^. Therefore, we anticipate more sequestration in deeper wells or aquifers that are confined by a seal layer as compared to shallow unconfined formations. Depending on the mineralogy of the aquifer, near-well regions may go through dissolution of minerals (especially carbonates), generating wormholes^46^ or enhancing preferrential flow pathways like faults and fractures^47^. On the other hand, some of the dissolved CO$_2$ may precipitate as carbonates in faults and fractures, in deeper subsurface^48,49^ as is evidenced in many outcrops^50,51^. Some of the gaseous CO$_2$ may enter into the vadose zone and the soil. Depending on the level of vegetation in the area, some of the CO$_2$ may get consumed by plants. Thus, The net proportion of sequestration from the initially stored CO$_2$ quantity will differ from site to site. A few case studies conducted under varying hydrogeological conditions should help draw some generally applicable lessons.
- *Impact on groundwater quality* Undisturbed naturally occuring groundwater can be usually found in chemical equilibrium with the solid phase (rock minerals) of the aquifer. However, when CO$_2$ is added to the aquifer, it dissolves in the groundwater leading to decreased pH (increased acidity) of the water, and thus disturbing the geochemical equilibrium. The subsequent geochemical reactions that get triggered between this acidified groundwater and the aquifer minerals (referred to as buffering) have the potential mobilize certain trace metals (including lead and arsenic). Studies have found that the concentrations of these mobilized trace metals do not reach upto or exceed the maximum permitted levels, except in rare cases where the aquifer is naturally rich in these minerals and already has elevated concentrations in the groundwater^52–54^.
- *Use of recycled CO* $_2$ In order to create a greater impact on climate change, it is crucial to obtain the injected CO$_2$ which is ‘captured’ from anthropogenic sources (such as coal-fired powerplants or other industries). CO$_2$ capturing technologies has been around for a few decades, but the economic challenges (high cost) have been at the forefront in inhibited the wide commercial implementation of these techniques^55^. However, the global push towards becoming carbon neutral has driven significant research and development of these technologies, including a few commercial-scale initiatives that are in the early stages^56^. Direct Air Capture of CO$_2$ is yet another challenging but critical field of upcoming technologies that work on capturing CO$_2$ directly from the atmosphere instead of a point source like a chemical plant. Utilization of recycled CO$_2$ is critical to achieving net-zero, and techno-economically efficient CO$_2$ capture methods are the first step in the direction.

## Conclusion

An analysis and review of the CO$_2$ utilization and storage potential of the Aqua Freed^®^and Aqua Gard^®^technologies demonstrates the benefit of using CO$_2$ for well rehabilitation and preventive maintenance in improving well performance and efficiency and reducing the carbon footprint. Thus, injecting CO$_2$ into the subsurface for well rehabilitation is yet another proven method for achieving CCUS and moving towards net-zero emissions. Typically, about 90% of the injected CO$_2$ is estimated to remain in the subsurface. CO$_2$-powered well rehabilitation if adopted widely as a CCUS technology has the capacity to store several megatonnes of CO$_2$ annually.

## Methods

### Field data collection

The loss during venting was estimated by placing a flowmeter at the vent and timing the venting process. For estimating the loss during flushing, the concentration of CO$_2$ was measured along time in the field as the groundwater was being flushed out during AF/AG treatment of 12 different wells processed during the course of this study. The procedural steps of measuring CO$_2$ concentrations in the field are as follows:

- Measure 100 mL of water in a graduated cylinder and transfer to flask.
- Pour in 1 packet of phenolphthalein indicator powder and let it dissolve.
- Use handheld, digital titrator equipped with a cartridge of 3.636 N.
- Swirl in drops of sodium hydroxide until the water turns a light hue of pink
- Take the number read on the digital titrator and multiply it by a suitable multiplier depending on the amount of water measured and the range desired.

In addition to the CO$_2$ concentrations, the discharge rate at which the well was flushed was also noted.

### Statistical data modeling

We use a linear mixed-effects regression approach to model the CO$_2$ concentration trends during the flushing phase of an AF/AG well event. A Linear mixed-effects (LME) model is an extension of the simple linear regression (SLR) model^29^. It has been found useful to adopt the LME approach when the collected data can be segregated into meaningful subgroups, and is widely applied to biological, social and healthcare research^28^. In the case of this study, the subgroups are the individual well data. The LME model jointly estimates well-specific regression coefficients along with the population-level regression coefficients. Thus, we derive the overall trend (across all well events) as well as well-specific trends (Table 1).

It is important to note that the LME approach is very different from estimating well-specific trends individually, and then separately estimating the overall trend. The LME model estimates the ‘distribution’ of well-specific random effects around the population-level fixed effects. Thus, the fixed effect parameters (slope and intercept) estimated by LME may differ from the slope and intercept values that a SLR model would yield.

Some of the advantages of using LME over SLR for data that can be summarized groupwise are as follows:

- LME uses the data much more efficiently making it better scalable to larger number of wells.
- Due to the above feature, it works well with unbalanced and smaller data sets (by avoiding convergence issues) and can utilize and accommodate missing observations (e.g. concentration measurements missing at certain time points.)
- LME jointly estimates population and subject-specific values and therefore is the more accurate (and interpretable) representation of repeated-measures data. It naturally takes into account the correlation between repeated scans from subjects. This is why it naturally prevents overfitting.

Since the concentration data of well events from dataset1 is exponential, natural logarithmic values of the concentration were used for our regression modeling, which is linear. Four regression models (3 different combinations of the LME and 1 SLR for comparison) were tested and cross-validated to find the one that best represents of the data.

Model choices:

1. LME: Both slope and intercept vary for both fixed and random effects
2. LME: Both slope and intercept vary for both fixed effects; only intercept varies for random effects
3. LME: Both slope and intercept vary for both fixed effects; only slope varies for random effects
4. SLR: Simple linear regression applied to concentration data of all well events from dataset1 combined (for comparison with LME)

Cross-validation is one of the most popular statistical techniques that is widely used model validation^57,58^. Essentially, cross-validation tells us how well would the selected model generalize to ‘unseen’ data. Details and results of model selection and cross-validation are provided in supplementary information. Model 2 was found to have the least ‘test’ error (meaning it generalizes the data best) and was selected for further analysis in the “Results” section.

All modeling was performed using the MATLAB software^59^. The code for this study is based on original code developed by Sharma^60,61^.

## Supplementary Information

Supplementary Information.

## Data Availability

The datasets generated during and/or analyzed during the current study are available from the corresponding author upon reasonable request.
