# Supplementary material for: Carbon utilization and storage through rehabilitation of groundwater wells
Source: Sci Rep. 2024 Jun 15;14:13838. doi: 10.1038/s41598-024-64135-z (PMC11637122; doi:10.1038/s41598-024-64135-z)
Supplement: Supplementary file 1 — Supplementary Information. [file 41598_2024_64135_MOESM1_ESM.pdf]

Supplementary Information: Carbon Utilization and Storage through Rehabilitation of Groundwater Wells

Vivek V. Patil* Gabriella Basso Steven Catania Christopher Catania
Timothy Ostapuk Robert Vince

*vivek.patil@wsp.com

The four linear regression model choices considered were:

1. LME: Both slope and intercept vary for both fixed and random effects
2. LME: Both slope and intercept vary for both fixed effects; only intercept varies for random effects
3. LME: Both slope and intercept vary for both fixed effects; only slope varies for random effects
4. SLR: Simple linear regression applied to concentration data of all well events from dataset1 combined (for comparison with LME)

Cross validation was used for model selection. Dataset1 (16 well events) was split into 80% training set and 20% test set. The training set was used for build the regression model and estimate the parameters. The test set was then used to determine how the estimated model fit would perform with unseen data. The data sets were shuffled after every iteration and new training and test sets were created before the next iteration. The total number of iterations was 1000. Table S1 presents the results of cross validation averaged over 1000 iterations.

Based on the cross validation results, model 1 and 2 can be shortlisted as the two models that perform much better for dataset1. Model 1 has the least training Mean Squared Error (MSE). However, model 2 has the least test MSE. This implies that while model 1 will do the best job at fitting a trend for dataset1, model 2 will do a better job at generalizing for unseen data. In other words, model 2 is better for avoiding overfitting. Since the aim of this regression exercise is to predict the CO_2 storage estimates and project those estimates over a larger database unseen by the model, Model 2 is the right choice for this study.

AIC and BIC are another set of statistics that are useful for model comparison and selection. In general, lower AIC and BIC values indicate better model fit. For both model 1 and 2, AIC and BIC values are comparable, and these are lower than AIC and BIC for models 3 and 4. P-values for all models are at least 2 orders of magnitude lower than 0.05 indicating that results of all model choices are statistically significant.

The fitted vs. observed plot and the residual plot are both graphical representations of the goodness of fit. As seen in Figure S1, these plots are comparable for models 1 and 2. The fitted vs. observed plots for models 1 and 2 show that the points randomly align along the diagonal (y=x) line, indicating that no systematic underprediction or overprediction by the models. The residual plots for model 1 and 2 show that all the residuals are randomly scattered around the horizontal (residual = 0) line, indicating that both the models are an acceptable choice for the data. On the other hand, the fitted vs. observed plot for model 4 (SLR) shows that the model overpredicts at lower concentration values and underpredicts at higher concentration values. This results proves that a linear mixed-effects (LME) regression approach is better suited for this dataset than a simple linear regression approach.

The final regression parameters and statistics for all model choices are presented in Table S2. Our choice of model in this study was model 2.

Table S1: Cross validation results. All values are averaged over 1000 iterations. Asterisk (*) indicates that results are statistically significant (p-value less than 0.05). MSE = Mean Squared Error. AIC = Akaike Information Criterion. BIC = Bayesian Information Criterion. Training set = 80% of dataset1. Test set = 20% of dataset1.

Model	MSE (Test)	MSE (Training)	AIC	BIC	Fixed Intercept	Fixed Slope
1	0.968	0.151	120.1	133.2	6.29*	-0.119*
2	0.759	0.224	122	130.7	6.267*	-0.091*
3	0.824	0.512	151.6	160.3	6.11*	-0.081*
4	0.796	0.577	151.3	155.6	6.079*	-0.075*

Table S2: Results from models applied to the entire dataset1. Asterisk (*) indicates that results are statistically significant (p-value less than 0.05). MSE = Mean Squared Error. AIC = Akaike Information Criterion. BIC = Bayesian Information Criterion.

Model	MSE	AIC	BIC	Fixed Intercept	Fixed Slope
1	0.146	149.9	164.3	6.294*	-0.12*
2	0.22	152.7	162.3	6.271*	-0.091*
3	0.574	195.2	204.8	6.077*	-0.075*
4	0.767			6.077*	-0.075*

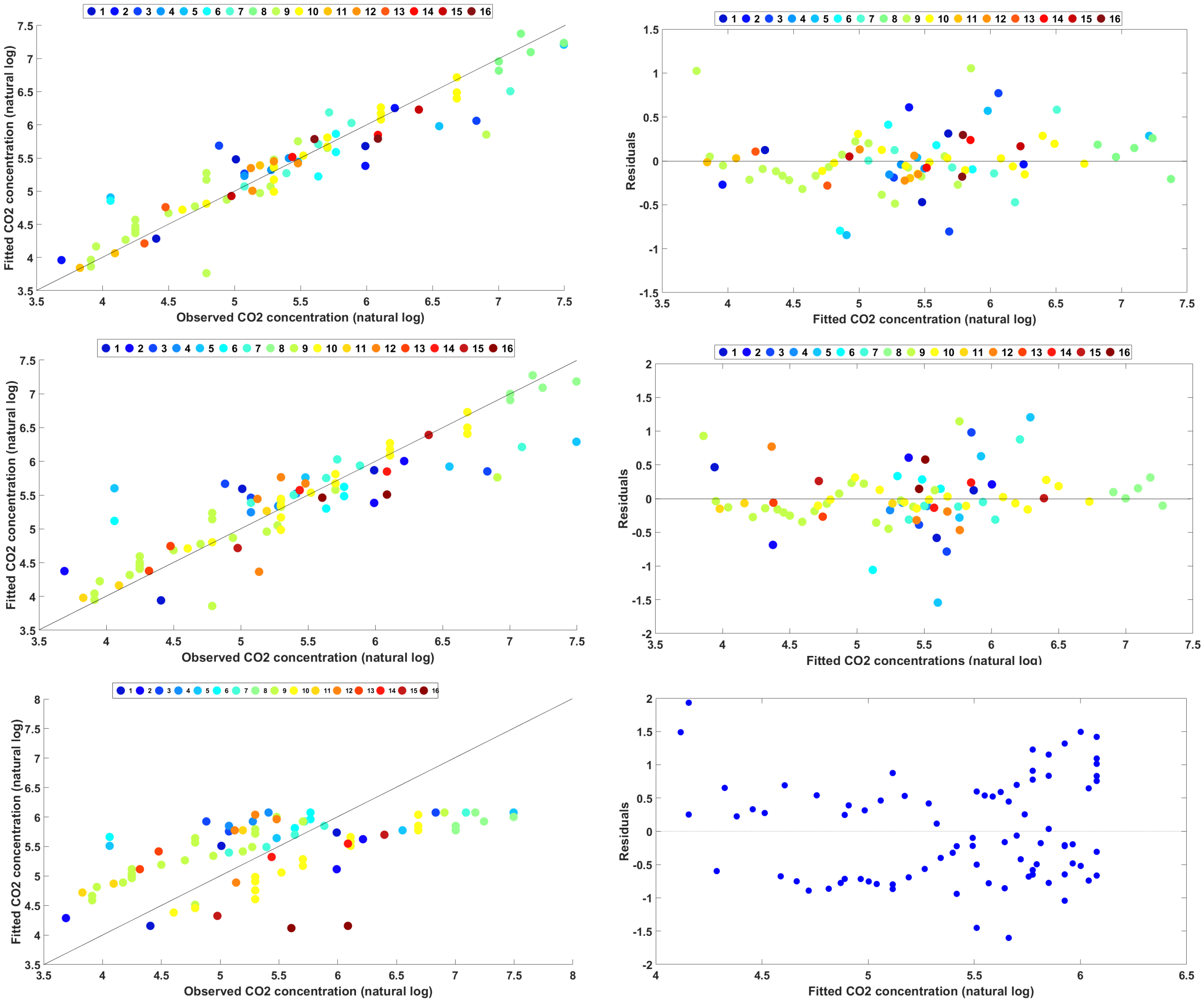

Figure S1: The goodness of fit demonstrated through predicted vs. observed plots (left column) and residual plots (right column). Row 1: Results for model 1 fit (LME); Row 2: Results for model 2 (LME); Row 3: Results for model 4 (SLR). Graphs color coded by the 16 wells from dataset1.
